# Plant Secondary Metabolites: An Opportunity for Circular Economy

**DOI:** 10.3390/molecules26020495

**Published:** 2021-01-18

**Authors:** Ilaria Chiocchio, Manuela Mandrone, Paola Tomasi, Lorenzo Marincich, Ferruccio Poli

**Affiliations:** Department of Pharmacy and Biotechnology, Alma Mater Studiorum—University of Bologna, Via Irnerio 42, 40126 Bologna, Italy; ilaria.chiocchio2@unibo.it (I.C.); paola.tomasi3@unibo.it (P.T.); lorenzo.marincich2@unibo.it (L.M.); ferruccio.poli@unibo.it (F.P.)

**Keywords:** circular economy, waste valorization, plant by-products, plant secondary metabolites, natural products, bioactivities

## Abstract

Moving toward a more sustainable development, a pivotal role is played by circular economy and a smarter waste management. Industrial wastes from plants offer a wide spectrum of possibilities for their valorization, still being enriched in high added-value molecules, such as secondary metabolites (SMs). The current review provides an overview of the most common SM classes (chemical structures, classification, biological activities) present in different plant waste/by-products and their potential use in various fields. A bibliographic survey was carried out, taking into account 99 research articles (from 2006 to 2020), summarizing all the information about waste type, its plant source, industrial sector of provenience, contained SMs, reported bioactivities, and proposals for its valorization. This survey highlighted that a great deal of the current publications are focused on the exploitation of plant wastes in human healthcare and food (including cosmetic, pharmaceutical, nutraceutical and food additives). However, as summarized in this review, plant SMs also possess an enormous potential for further uses. Accordingly, an increasing number of investigations on neglected plant matrices and their use in areas such as veterinary science or agriculture are expected, considering also the need to implement “greener” practices in the latter sector.

## 1. Introduction

According to the United Nations [1], the global population is expected to increase from 7.7 billion (2019) to 9.7 billion in 2050. This prospect raises several concerns about the global consumption of biomass, fossil fuels, metals, and minerals, which should double [2], and annual waste production, following the current trend, will increase by 70% in the next 40 years [3]. Undoubtedly, these premises challenge the move toward a more sustainable development.

In this scenario, wastes and by-products from the food and agricultural industries are gaining international attention, not only for the issues associated with pollution, but also to overcome the paradox of 820 million people suffering from hunger and malnutrition while others are dealing with food over-consumption and related diseases, together with increasing food waste production [4,5]. Moreover, food waste and overproduction imply unnecessary exploitation of the environment and natural resources such as carbon, water, and land. The land footprint estimated by the FAO (Food and Agriculture Organization of the United Nations) in 2013 [6] revealed that almost 30% of the world’s agricultural lands are used to produce food that is ultimately lost or wasted, determining additional pollution and greenhouse gases emissions to no actual purpose. Furthermore, food overproduction and bad waste management also have a negative impact on the economy, with money loss at different levels of the supply chain. Conversely, the reduction of food loss and waste generation has the potential to generate considerable economic value.

**Figure 1:**
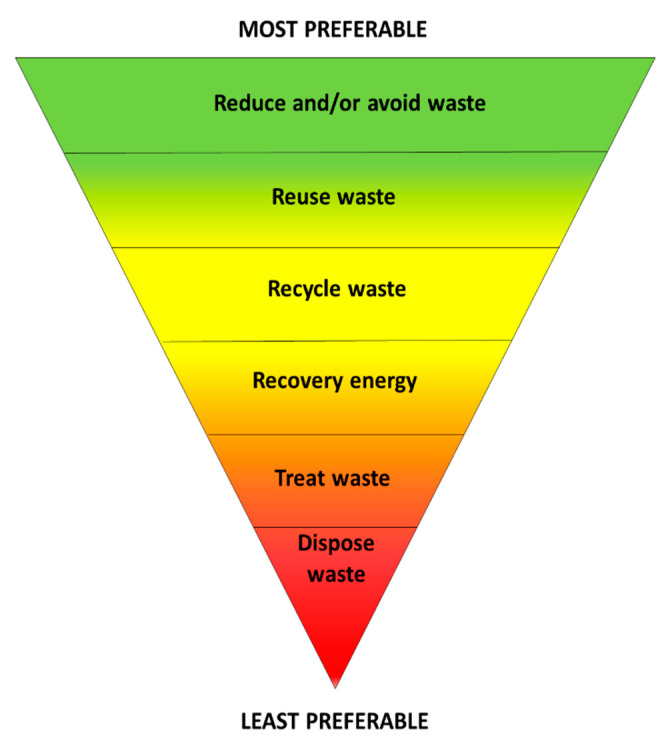
Scheme of the “waste hierarchy” proposed by the EPA [8].

ReFED (Rethink Food waste through Economics and Data), a multi-stakeholder nonprofit committed to reducing food waste, identified 27 solutions, which were grouped into three categories: prevention, recovery, and recycling [7]. Although the associated incomes cannot be generalized for all countries, following these solutions, USD 100 billion is expected over 10 years in U.S. According to this program, the solutions focused on preventing waste production account for over 75% of the total, while 23% relate to waste recovery, and only 2% to recycling [5]. This proposal is consistent with the EPA (Environmental Protection Agency) “waste hierarchy,” which grades waste prevention as the most preferable option (Figure 1) [8].

In this scenario, the idea of circular economy took shape, promoting the shift from a linear economic scheme of “take-make-use-dispose” to a circular model, employing reuse, sharing, repair, refurbishment, remanufacturing, and recycling to create a closed-loop system, attempting to minimize the use of resource inputs and the generation of waste, pollution, and carbon emissions [9,10]. In Europe, the most relevant strategy launched in order to attain a sustainable development is the Circular Economy Action Plan, according to which waste materials and energy become input for other industrial processes or regenerative resources for nature (i.e., compost) [11]. In order to facilitate and stimulate the implementation of these guidelines, the scientific community is paying increasing attention to the valorization of industrial and agricultural wastes and by-products, in particular, those derived from plants. These neglected matrices are often manufactured for biofuel production, such as methane or ethanol [12]. However, according to the proposed “waste hierarchy” (Figure 1), energy recovery is a less preferable strategy of waste management, compared to others. Moreover, although biofuel is an alternative to petroleum-derived fuels, its sustainability is quite controversial [13,14].

Actually, the high added-value molecules still contained in plant wastes and by-products offer a wide spectrum of possibilities for their valorization and reuse, as foreseen by circular economy [15,16]. For example, from agri-food wastes it is still possible to extract macromolecules such as nucleic acids [17], pectins [18], cellulose material [19], and enzymes such as bromelain, which is derived from pineapple residues and extensively used as a pharmaceutical and meat tenderizer [20]. Primary metabolites (i.e., organic acids, amino acids, carbohydrates) can also be obtained from plant waste material and used for different purposes [21]. Plants in particular produce secondary metabolites (SMs), which are not directly involved in the basic functions of growth, development, and reproduction of the organism, but are essential for long-term survival and play multiple roles, including defense against predators or attraction of pollinators [22]. SMs are endowed with numerous biological activities, making them also extremely important for human health and well-being. Moreover, due to their chemical and biological properties, SMs have also found application in many other fields, serving as pigments, cosmetics, antifeedants and so on [23].

SMs are usually classified according to their biosynthetic pathways in three principal groups: phenolics, terpenes, and alkaloids [24]. These phytochemicals are characterized by enormous chemical and biological diversity and, in addition to being species-specific and organ-specific, their production depends on many biotic and abiotic factors.

One of the most common examples of SM recovery from plant by-products is from fruit peel generated from industrial processes [25], as in the case of citrus peel, from which essential oils [26] as well as phenolic compounds [27] are extracted. Phenolic acids, flavonols, and catecholamines are obtained also from banana peel [28], while carotenoids such as lycopene are usually obtained from tomato peel and other industrial tomato by-products [29].

Interestingly, SMs are potentially present in all plant organs, offering several possibilities for the valorization of wastes from plant cultivation. In fact, in the agricultural sector, only a few plant organs are harvested and fully consumed, generating numerous wastes at different levels of the supply chain. In this context, scientific investigations aimed at identifying the bioactive compounds contained in these neglected matrices play a pivotal role in laying the basis for their valorization.

Aimed at facilitating and encouraging research projects focused on plant wastes/by-products and circular economy implementation, the current review provides an overview of the most common SMs present in plant matrices, their classification and bioactivities, and the consequent potential application in different fields of the waste material containing these compounds. Consequently, in this work 99 publications (from 2006 to 2020) focused on plant waste/by-product valorization were reviewed and tabulated in order to schematize the state of the art on this topic and offer the opportunity to easily extrapolate information for the design of new studies on neglected plant material and its reuse in a circular economy perspective.

## 2. Polyphenols

### 2.1. Chemical Structure and Classification

**Figure 2:**
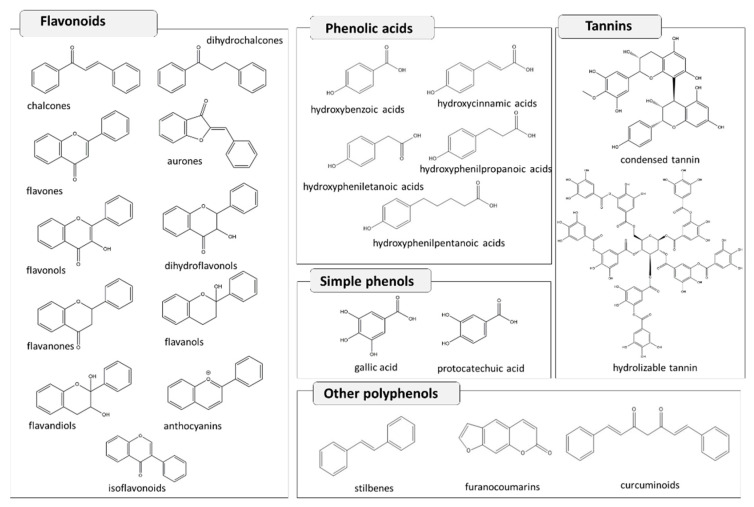
Examples of polyphenols, basic nucleus and classification.

Polyphenols are one of the largest and most complex classes of SMs produced by plants, derived from shikimate biosynthesis pathway, which provides precursors for aromatic molecules. Based on the biosynthetic pathway, the number of aromatic rings, carbon atoms, and hydroxyl groups, they are divided into different sub-classes such as: simple phenols, phenolic acids, flavonoids and tannins (Figure 2).

Simple phenols are constituted by a single benzenic ring (C_6_) linked to a hydroxyl group such as resorcinol, orcinol, catechol, guaiacol, hydroquinone, and phloroglucinol.

Phenolic acids present a carboxylic group among the substituents on the benzene ring, and they are generally divided into benzoic acid derivates (C_6_-C_1_) (i.e., gallic acid, vanillic acid, syringic acid) and hydroxycinnamic derivates (C_6_-C_3_) (i.e., caffeic acid, ferulic acid and coumaric acid).

Flavonoids (C_6_-C_3_-C_6_) are generally constituted by two benzenic rings (A ring and B ring) linked by a chain made of three carbons, often condensed into a pyranic ring (C ring). Given their complexity, flavonoids are divided into other sub-classes such as: chalcones, dihydrochalcones, aurones, flavones, flavonols, dihydroflavonol, flavanones, flavanol, flavandiol or leucoanthocyanidin, anthocyanidin, isoflavonoids. Moreover, flavonoids exist in the aglycone form or as glycoside derivatives [30].

Tannins are high molecular weight polyphenols, usually distinct into condensed tannins (proanthocyanidins), which are polymers constituted by flavonoids units, and hydrolysable tannins characterized by a monosaccharide, normally D-glucose, esterified with one or more molecules of gallic acid (gallotannins), or ellagic acid (ellagitannins). Hydrolysable tannins are more labile in acid, alkali, or hot water than condensed tannins [30].

### 2.2. Distribution in Plants and Biological Activities

Polyphenols are widely distributed in all plant organs [31]. In general, phenolic acids are found in seeds, leaves, roots, and stems [32], while flavonoids are prominently found in aerial parts, and tannins in roots, bark, and seeds [33].

This heterogeneous group of SMs plays different roles in plants. For instance, flavonoids confer color to the flowers, attracting insects and promoting pollination [34]; some polyphenols are deterrents for herbivores [35], others are very important for UV-protections and for their antioxidant properties [36,37].

Polyphenols, especially flavonoids, are considered important molecules for the human diet and, consequently, are often proposed as ingredients in food supplements and nutraceuticals. In fact, in addition to being antioxidant agents, they are endowed with several other biological activities [38]. For instance, flavonoids were proven active as anti-inflammatory [39], anticancer [40,41], antihypertensive [42], microcirculation improving [43], and hypolipidemic agents [44]. They proved also interesting as active ingredients in the cosmetic field [45,46,47] and as natural dyes [48].

Phenolic acids are naturally found in fruits and vegetables, and are endowed with a wide spectrum of bioactivities such as: antidepressant [49], antihypertensive [50], anti-inflammatory [51], neuroprotective [51], antihyperglycemic [52], anticancer and anti-diarrheal [53].

Regarding tannins, they are used in the veterinary field as anthelmintic and antimicrobial agents [54,55], as well as in the leather industry for their tanning properties [56]. However, tannins should be used carefully, since in addition to their health-promoting properties, some toxic effects have also been reported [57,58]. Moreover, although tannin-rich ingredients are often added to ruminants’ feed, there is still a lack of information about the interaction between hydrolysable tannins and ruminants’ gastrointestinal microbiota. The fate of hydrolysable tannin metabolites derived from gastrointestinal microbial activity in the animal is still underexplored. It is known that some metabolites derived from hydrolysable tannins (i.e., pyrogallol) have adverse effects on gastrointestinal microbiota and the host animal [59]. These data point out the need of deeper investigation on tannin uses and effects, taking into account that risks and benefits depend on the specific situation and concentration used.

### 2.3. Polyphenols from Agro-Industrial Wastes and By-Products

Polyphenols being present in a wide spectrum of plant organs, they are easily found in numerous agro-industrial wastes/by-products, offering several possibilities for their valorization. First of all, an excellent polyphenol source is represented by the main by-products of wine production, namely: pomace, skins, and seeds. In particular, molasses seeds have a high flavonoids content, and molasses pomace is rich in tannins [60]. Grape pomace contains gallic acid, syringic acid, vanillic acid, catechin, isoquercitrin, and epicatechin [61]. The presence of these antioxidant metabolites [60,61] makes the wine by-products useful additives for ruminant feed [62]. In addition to the antioxidant potential, pomace also shows anti-cholesterol activity [63], while tannins from pomace are also used as wood adhesive [64].

Another polyphenol source is olive pomace [65], a by-product of the olive oil supply chain. This matrix is rich in tyrosol and its derivatives, and it also contains flavonoids such as rutin, apigenin, luteolin, taxifolin, diosmetin, and quercetin, and phenolic acids such as cinnamic, p-coumaric, caffeic, vanillic, and ferulic acid [66,67]. Due to its polyphenol content, olive pomace is also added to ruminant feed [68].

Moreover, olive wastewater contains polyphenols such as hydroxytyrosol [69,70], whose antioxidant and antibacterial properties make it a useful ingredient for cosmetic formulations [71].

Juice industry by-products such as pomace, skins, and seeds are another potential source of polyphenols. Among them, apple pomace is an example [72], since it contains catechin, epicatechin, chlorogenic acid, procyanidin B2, phlorizin, and gallic acid [73].

Among by-products derived from juice production, noteworthy are also strawberries, blueberries, carrots, and pears. In particular, black currants and chokeberries are the richest in anthocyanins, which are suitable ingredients for animal feed [74,75] and textile dyes [76].

Polyphenol-rich wastes derive also from agricultural remains after harvesting [75], canning, and liquor industries [77].

Regarding the aromatic herb industry, basil, sage, and rosemary generate several kind of wastes resulting from pruning, packaging, or distillation processes. It was proved that wastewater from the distillation of these herbs contains important metabolites such as glycosylated flavonoids and caffeic acid derivatives, above all, rosmarinic acid [78]. Wastewater generated from aescin (a saponin from horse chestnuts) production contains kaempferol and quercetin [79].

By-products containing polyphenols are also generated from the production of soluble coffee. For example, coffee grounds, which is the principal by-product, contain many polyphenols such as condensed tannins [80], chlorogenic acid, p-coumaric acid, ferulic acid, rutin, naringin, resveratrol [81]. By virtue of these SMs, coffee grounds proved active to inhibit seed germination [82]. Another by-product from this supply chain is the silverskin, a thin tegument covering coffee beans that is removed in the roasting process. Silverskin is rich in chlorogenic acid derivates such as dicaffeoylquinic acids and feruloylquinic acids [83,84,85].

Regarding the agricultural sector, chestnuts wastes from *Castanea sativa* Miller, such as spiny burs, are noteworthy for their polyphenol content [86,87,88,89], including gallic acid and ellagic acid derivatives, together with glycosylated flavonoid [90,91]. By-products derived from chestnut flour production such as chestnut peels are sources of polyphenols, in particular, tannins [86,88,92]. For this reason, chestnut spiny burs and peels are valuable as natural antioxidants and often used in animal feed [86]. Also bud pomace of *C. sativa* contains cinnamic acids, benzoic acids, flavonols, and catechin [93]. Regarding *C. sativa* cultivation for wood production, the principal waste is the bark, which is also an important source of tannins [94], similar to other tree barks [95].

Several other polyphenol-rich by-products are known, such as *Cocoa* shell, the waste of chocolate production, which contains catechin, epicatechin, and gallic acid [96]; the waste of black tea, which shows antioxidant and antimicrobial activity [97]; melon peels, which contain a great amount of polyphenols [98]; larch bark, a source of proanthocyanidin B7, a well-known antioxidant [99]; and maize bran containing ferulic acid [100].

## 3. Terpenes

### 3.1. Chemical Structure and Classification

**Figure 3:**
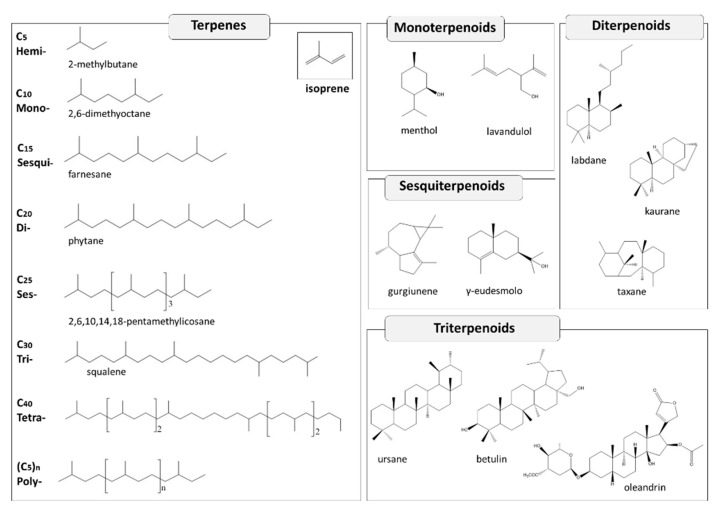
Some examples of terpene chemical structures and classification according to the number of isoprene units contained in the structure.

Terpenes, also termed terpenoids or isoprenoids, constitute a large family of natural products extremely diversified in their structure, functions, and properties. Terpenes derive from mevalonic acid biosynthetic pathway. However, since their decomposition generates isoprene units (C_5_), this compound has been defined as terpene’s basic constituent. For this reason, these SMs are classified based on the number of isoprene units present in their structure, and generally condensed head-to-tail. Following this rule they are divided into hemiterpenes (C_5_H_8_), monoterpenes (C_5_H_8_)_2_, sesquiterpenes (C_5_H_8_)_3_, diterpenes (C_5_H_8_)_4_, sesterterpenes (C_5_H_8_)_5_, triterpenes (C_5_H_8_)_6_, etc. [101] (Figure 3).

The broader term of terpenoids is applied to terpene-related molecules that, in addiction to isoprene units, contain other substituents, for instance, oxygenated functional groups [102]. Many other natural substances, such as alkaloids, phenolics, and vitamins, despite deriving from biosynthetic pathways other than mevalonate (i.e., acetate or shikimate), are sometimes classified as meroterpenoids since they present isoprenic moieties in their structure.

Hemiterpenes are quite rare; one example is isoprene itself, which is a volatile compound present in several plants, especially trees. Monoterpenes are generally responsible for essential oils fragrance, some examples are: limonene, borneol, camphor, pinene, cineole, menthol, thymol, carvacrol. Diterpenes have as a precursor geranylgeranyl diphosphate; examples are phytol, which forms the lateral chains of chlorophylls, and taxol isolated from *Taxus brevifolia* Nutt, which is an important natural antitumor agent. Other renowned diterpenes are labdanes, which are the major components of the resin produced by plants of the Cistaceae family [103], and the gibberellins, important phytohormones regulating plant growth and development [104]. Squalene is triterpenes’ precursor, deriving from numerous compounds, including tetracyclic triterpenes like dammarenes, and pentacyclic triterpenes like lupanes and oleanes. The latter are often found in saponin skeletons, where they are glycosylated with one or more sugar moieties. Saponins are phytogenic biosurfactants, which induce foam formation in aqueous solution, reducing the viscosity of heavy crude oil-in-water emulsions [105]. Triterpenic saponins are found in numerous plants, the most renowned among them are *Saponaria officinalis* L. (soapwort), *Quillaja saponaria* Molina, and *Gypsophila arrostii* Guss. [106], which were, in fact, traditionally used for their soap properties. Saponins are classified on the basis of the differences occurring in their aglycone structure or sugar moiety. For example, on the basis of the sugar moiety they are classified into mono, bi, and tridesmosidic. The type of aglycone and the number of linked sugar residues determine the foam properties and the entity of adsorption at the interface [107].

Steroids can be considered modified triterpenes containing the lanosterol tetracyclic system without the two methyl substituents in C-4 and C-14 positions. Cholesterol is the basic structure of this class. They can be found also as saponins (steroidal saponins), such as cardioactive glycosides.

Tetraterpenes include carotenoids, while high molecular weight isoprenoid polymers (higher than C40) are found in natural rubbers produced, for example, by trees belonging to the Euphorbiaceae and Sapotaceae families [108].

### 3.2. Distribution in Plants and Biological Activities

Terpenes may serve a wide spectrum of functions in plants, including attracting pollinators or protecting injured tissues from herbivores, insects, and parasites attack. According to this biological role various monoterpenes are toxic to insects [109], fungi [110], and bacteria [111]. In addition, steroidal saponins such as cardenolides are toxic to many animals through their inhibition of Na+/K+-ATPases. However, the same property makes them useful as therapeutic agents, in carefully regulated doses, to slow down and strengthen the heartbeat.

Due to their numerous bioactivities, terpenes find various applications in several industrial sectors, such as pharmaceutical, food, cosmetic, perfumery, and agricultural, and are used as drugs, food supplements, flavors, fragrances, biopesticides, etc.

First of all, the peculiar fragrance of many monoterpenes, present principally in aromatic plant essential oils, makes this class of compounds extremely important for food, aromatherapy, and perfumes. In addition to the fragrance, monoterpenes from essential oils, such as thymol, thymine, and carvacrol (found principally in plants belonging to the Lamiaceae family) proved interesting also for their numerous biological activities, for instance, their potential in the treatment of disorders affecting respiratory, nervous, and cardiovascular systems, and as antimicrobial and antioxidant agents [112].

Also labdane-type diterpenes are useful for the perfume industry, finding application as fixatives in high-end perfumes. Specifically, a fixative is a material with low volatility that provides long-term scent, aids in mixing with the other materials, and extends the shelf life of the perfume. The resin obtained from some plants of the Cistaceae family is one of the most common sources of labdane diterpenes used as perfume fixatives [113].

Saponins are extremely important for the food industry. In fact, many of the processed foods, including baked goods, ice creams, sauces, desserts, and drinks, contain dispersions such as emulsions and foams used to stabilize, determine, and control texture and rheological properties of these products. Saponins, due to their amphiphilic properties, proved to stabilize food emulsions with less sensitivity to pH, ionic strength, and high temperatures (up to 90 °C) than currently used emulsifiers [114]. Moreover, the increasing consumer demand for plant-based and sustainable emulsifiers and foaming agents makes saponins much requested for the food industry nowadays.

Despite saponins being toxic at high dosages, small quantities have been approved as food additives. The two main commercial sources are *Q. saponaria* from Chile, whose saponins have a prominently triterpenoid structure, and *Yucca schidigera* Roezl ex Ortgies from Mexico, which contains saponins with a steroid structure [106].

Moreover, saponins at low dosage also showed numerous biological activities important for human healthcare, which are summarized in several review papers [115,116,117]; most relevant among them are the anticancer, cholesterol-lowering, and antiviral properties [118,119,120,121]. Some examples of the therapeutic potential of triterpenic saponins are provided by boswellic and betulinic acids. In fact, the extracts of the resin obtained from incense trees (*Boswellia serrata* Roxb.), containing the pentacyclic triterpenoid boswellic acid, have been employed as an anti-inflammatory drug [122], and the clinical trials on gum-resin from *B. serrata* have shown an improvement in the symptoms in patients with osteoarthritis and rheumatoid arthritis [123,124]. Betulinic acid, a naturally occurring pentacyclic triterpene, exhibited a high variety of biological activities [125], including potent antiviral effects [126].

Moreover, saponins are antimicrobial agents active also against bacteria and fungi invading plants [127,128]. The mechanism of these activities is likely based on saponins’ ability to form complexes with sterols present in the membrane of microorganisms and to cause, consequently, membrane perturbation [129,130]. Saponins also exert insecticidal [131] and molluscicidal [132] activities, as well as allelopathic activity toward different plant species [133]. These properties, together with their biological role in plant defense, confer to saponins an enormous potential as natural biopesticides useful for “green” agriculture practices. For example, Trdá et al. [134] found that saponin aescin, in addition to its antifungal effect against crop pathogens, is also able to activate plant immunity (in two different plant species) and to provide salicylic acid-dependent resistance against both fungal and bacterial pathogens.

In the pharmaceutical industry, terpenes are used as excipients to enhance skin penetration of active principles [135] and as therapeutic agents endowed with numerous bioactivities including, as mentioned above, chemo-preventive, antimicrobial, antifungal, antiviral, antihyperglycemic, analgesic, anti-inflammatory, and antiparasitic activities [110,111,118,122]. Among the pharmaceuticals, the anticancer paclitaxel and antimalarial artemisinin are two of the most renowned terpene-based drugs.

### 3.3. Terpenes and Terpenoids from Agro-Industrial Wastes and By-Products

Due to their numerous bioactivities, terpenes are particularly interesting in the context of waste requalification. Monoterpenes such as thymol and carvacrol are still present in discrete amounts in several by-products derived both from essential oil distillation and from the harvesting of some aromatic plants. For instance, the solid waste residues left after the distillation of leaves and stems of Mexican oregano (*Poliomintha longiflora* A. Gray) contain thymol and carvacrol, and are, as a result, endowed with antimicrobial activity [136]. Similarly, the monoterpenes limonene and nerol were found in fennel (*Foeniculum vulgare* Mill.) horticultural wastes [137].

The inedible part (stones, husks, kernels, seeds) from the fruit processing supply chain constitutes a huge portion of the consequent solid waste, which remains underexploited. For example, about one-third of citrus fruit production is industrially processed, with more than 80% used for orange juice production, which generates a huge amount of peel waste [138]. Orange essential oil mostly contains the monoterpene d-limonene (3.8% of orange peel dry weight) [139,140]. This molecule has been used as an ingredient in bio-based functional food, as preservatives for food [141], as well as in cosmetics and aromatherapy massage [142,143].

Moreover, the presence of d-limonene, an anti-fungal and antibacterial agent, makes orange oil a useful ingredient also for bio-pesticide formulations [144]. Finally, it is interesting to notice that waste orange peel, in addition to d-limonene, contains also other bioactive terpenes like linalool, and myrcene [145]. Besides orange peel, these terpenes can be found also in the peel of other citrus fruits such as lemon and several lime species [146], providing a good basis for the exploitation of this kind of wastes.

Regarding diterpenes, more than one million tons a year [147] of residue is produced after steam distillation of pine resin to recover the volatile fraction called turpentine. The consequent by-product is the gum rosin, which is a mixture of resin acids (90–95%) and other neutral compounds. The resin acids, most of which are isomers of each other, can be classified into two main categories: abietic-type (including abietic, neoabietic, palustric, and levopimaric), and pimaric-type (including pimaric, isopimaric, and sandaracopimaric) [148]. Gum rosin is a high-value-added residue, in fact, it is a natural alternative to fossil-based polymers obtained from the heating and evaporation of pine resin [149], as well as a producer of organocatalysts to promote complicated asymmetric industrial synthesis [150].

Several waste matrices contain saponins, which offer an enormous potential for their valorization. An example is provided by sisal (*Agave sisalana* Perrine), which is the main hard fiber produced worldwide. From its leaves, only the hard fibers (3–5% of total weight) are removed. The remaining 95–97% of the biomass is considered sisal waste, although it contains steroidal saponins, potentially useful for foods, cosmetics, and pharmaceuticals formulations, as well as for soil bioremediation [151].

Saponins from onion skin were found useful as a new natural emulsifier to formulate oil-in-water nanoemulsions by a high-pressure homogenizer [152].

Triterpenic acids such as oleanolic, betulinic, and ursolic acids provide another example of high-value terpenes that can be extracted from agricultural wastes prior to burning for energy production.

For example, oleanolic acid is found in agroforestry waste streams, such as in olive trees (*Olea europaea* L.), from which tons of wastes and by-products are generated on annual basis, including olive wood and leaves, cake, pomace, kernel, paste. During the production process, assuming a maximum content up to 3.1% of oleanolic acid in the leaves of *O. europaea*, a large amount of this high-value compound can potentially be extracted, contributing to the integrated valorization of the olive oil production chain [153]. In this context, the terpenes contained in the ethanol extract of olive milled residue showed anti-allergic activity on the cell line of rat basophilic leukemia, supporting the potential valorization of this other olive by-product [154].

*Humulus lupulus* L. (hops) flowers are used to preserve and give flavor to beer, while hops leaves are usually discarded as a waste. However, hops leaves contain β-caryophyllene, phytol, fatty acids, terpenes, and C_5_-ring bitter compounds, and the oil obtained from the leaves contains bitter acids, in particular cohumulinic, dehydrocohumulinic, and humulinic acid, possessing antibacterial activity [155].

Melon (*Cucumis melo* L.) is one of the most popular fruit cultivated in tropical countries and is industrially processed to obtain a wide spectrum of products such as juices, jams, dehydrated pulp, and salads or snacks, with consequent generation of a large number of by-products. However, these matrices (prominently pulp, seed, and peel) are still a good source of carotenoids (C_40_ tetraterpenoid pigments) like β-carotene, lutein, β-cryptoxanthin, phytoene, violaxanthin, neoxanthin, and zeaxanthin [156]. These substances are used to develop health-promoting functional food since they play an important role in eye photoprotection (provitamin A), improving immune functions, and preventing chronic diseases [157].

## 4. Alkaloids

### 4.1. Chemical Structure and Classification

A large number (more than 10,000 molecules) of plant SMs are classified as alkaloids [158]. The presence of nitrogen in the structure is the peculiar chemical feature of alkaloids. However, due to the huge structural diversity, alkaloid classification is extremely challenging. More recent classifications are based on carbon skeletons and/or biochemical precursor. However, this requires compromises in borderline cases, for example, the alkaloid nicotine (from *Nicotiana* spp.), which contains a pyridine fragment from nicotinamide and a pyrrolidine part from ornithine, could be correctly assigned to two different classes [108].

**Figure 4:**
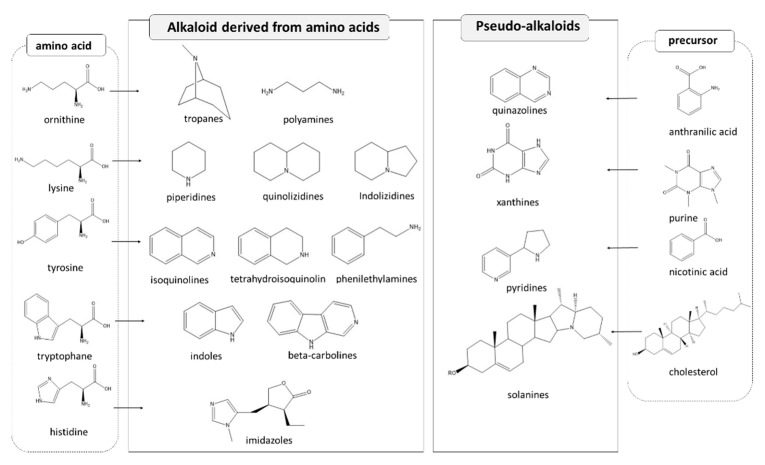
Examples of alkaloids (basic skeletons) and their precursors.

Historically, alkaloids have been defined as metabolites containing one or more nitrogen atom(s) within heterocyclic ring(s) [159]. However, N-containing compounds where the N atom is not heterocyclic, such as hordenine, ephedrine, colchicine and capsaicin, were further included into this SM group and classified as proto-alkaloids or amino-alkaloids. For this reason heterocyclic N-containing compounds are often regarded as “true alkaloids.” Since “true alkaloids” biosynthetically derive from amino acids, they are classified on the basis of the biogenetic origin (Figure 4).

In particular, the majority of them derive from ornithine, leucine, lysine, tyrosine, tryptophan, histidine, and phenylalanine. More specifically, pyrrole alkaloids derive from leucine; pyrrolidine, tropane, and pyrrolizidine alkaloids from ornithine; quinolizidine, and indolizidine alkaloids derive from lysine; catecholamines, isoquinoline, tetrahydroisoquinoline, and benzyltetrahydroisoquinoline alkaloids originate from tyrosine; indolamines, indole, carboline, quinoline, pyrrolindole and ergot alkaloids come from tryptophan; and imidazole alkaloids from histidine.

As the “true alkaloids,” proto-alkaloids derive from amino acids, and on this basis they are subsequently divided into phenylethylamino alkaloids, pyrrolizidine alkaloids, terpenoid indole alkaloids.

Following this classification criterion, another class of alkaloid, namely “pseudo-alkaloids,” was constituted, including compounds that do not originate from amino acids, while having a nitrogen atom inserted into the molecule by transamination or amination reactions (Figure 4). This letter class includes aromatic alkaloids, ephedra alkaloids, purine alkaloids, sesquiterpene alkaloids such as isoprenoid alkaloids including mono- (from geraniol), di- (from geranylgeranyl-PP), and triterpene (from cholesterol) derivatives, these latter called steroidal alkaloids [158].

Regarding steroidal “pseudo-alkaloids,” they are often glycosylated (glycoalkaloids). These peculiar compounds are produced in more than 350 plant species, mainly from Solanaceae and Liliaceae families [160]. They consist of a C_27_ cholestane skeleton (aglycone), where the –OH in position -3 is glycosylated by one to five monosaccharides, such as D-glucose, D-galactose, D-xylose and L-rhamnose.

Other peculiar alkaloids are the polyamine alkaloids (derivatives of putrescine, spermidine, and spermine), peptide and cyclopeptide alkaloids [161,162].

### 4.2. Distribution in Plants and Biological Activities

Alkaloids are an enormous group of phytochemicals of ecological importance, and possess a number of toxicological, pharmacological, nutritional, and cosmetic activities. Alkaloids are extremely abundant in flowering plants (Angiospermae), with a wide distribution in all organs such as leaves, flowers, seeds, roots, stems, fruits, bark, and bulbs. However, the presence and the distribution of these metabolites depend on the phase of plant life cycle, and strongly vary according to plant species, which produce different types of alkaloids, accumulated in various organs [163,164].

Alkaloids play numerous roles in plants, due to their involvement in defense [165,166,167], allelopathy [168], seed dispersal, and pollinator attraction [169,170]. Consistent with their defensive role, the highest alkaloid content is often found in plant reproductive organs [171]. In fact, many alkaloids are toxic to different organisms, protecting plants from pathogens and preventing non-specialist herbivore grazing [165,167]. In contrast, other alkaloids are essential for plant–pollinator interactions, increasing the number of pollinator visits, thus favoring plant reproduction [169,170].

Alkaloids have been historically used as drugs, and they remain very important in this context [172]; an example is provided by morphine from poppy straw, which is one of the most used analgesics today.

The biological activities of the principal sub-classes of alkaloids have been well summarized by Debnath et al. [159]. To cite some examples, quinine and quinidine, two quinolone alkaloids obtained from the bark of *Cinchona officinalis* L. (Rubiaceae), are very important historically used antimalarial drugs [173]; ephedrine, an adrenergic amine from the plants of genus *Ephedra* (Ephedraceae family), is used in many pharmaceutical preparations such as bronchodilators for asthmatic and allergic conditions, and to prevent low blood pressure during spinal anesthesia [174]; vinblastine and vincristine, two indole alkaloids extracted from *Catharanthus roseus* (L.) G. Don (Apocynaceae) [175], are renowned antitumor drugs.

Many other alkaloids have been studied for their promising bioactivities, for example, catuabine, a tropane alkaloid obtained from the bark of *Trichilia catigua* A. Juss. (Meliaceae) is endowed with antidepressant-like effects on the forced swim model of depression in mice and rats [176]; berberine, occurring in roots and stem-bark of different species of *Berberis* (Berberidaceae), showed anti-diabetic effect in rodent models of insulin resistance [177], and anti-hypertensive, anti-inflammatory, antioxidant, antidepressant, hepatoprotective activity, and anti-cancer activity [178,179].

Moreover, since some alkaloids possess psychotropic properties, they have found a role in social and ceremonial activities, as well as being important for popular spices and drinks, like the alkaloid caffeine, which is present in coffee [180]. Specifically, caffeine is a methyl-xanthine alkaloid, and its most important biological sources are *Coffea arabica* L. and *Camelia sinensis* (L.) Kuntze (leaves) (Theaceae). Caffeine is the most widely consumed stimulant drug in the world. It is also used in cold medications, analgesics, slimming agents, and cosmetics.

Moreover, consistent with their defense role, many alkaloids exhibit insecticidal [181,182], and fungicidal activity. For example, a piperidine alkaloid, pipernonaline, isolated from the hexane fraction of *Piper longum* L., showed potent fungicidal activity against the phytopathogen *Puccinia recondita* [183]; *Coptis japonica* (Thunb.) Makino extracts and the contained alkaloids (isoquinoline alkaloids, berberine chloride, palmatine iodide, and coptisine chloride) expressed fungicidal activities against several phytophatogens, namely: *Botrytis cineria*, *Erysiphe graminis*, *Phytophthora infestans*, *Puccinia recondita*, *Pyricularia grisea*, and *Rhizoctonia solani* in in vivo plant models [184].

Relevant in this context are also the alkaloids produced by the Solanaceae family, which have an enormous potential to deliver new chemicals for crop protection. In fact, more and more of these compounds, or mixtures of them, are being identified as pest control agents, especially against insects, fungi, and mites [185]. The Solanaceae family belongs to the most important plant taxa, particularly in terms of food production (i.e., tomatoes and potatoes). Tomato and potato are the best known and most widely used plants of this group, and they constitutively synthesize low levels of many different glycoalkaloids. These natural toxicants (stress metabolites) have insecticidal and fungicidal properties and, since naturally occurring pesticides are often biosynthesized when plants are under stress, injuries on plant tissues promote the synthesis of higher concentrations of these compounds.

### 4.3. Alkaloids from Agro-Industrial Wastes and By-Products

The neglected matrices containing alkaloids have a high requalification potential by virtue of the numerous bioactivities possessed by these metabolites.

For instance, caffeine, a methyl-xanthine alkaloid, besides being an important ingredient for energy drink industry, it is also relevant for cosmetics. Caffeine is, in fact, used for cellulitis reduction [186,187], and to prevent skin aging through both antioxidant activity and inhibition of skin remodeling enzymes [188].

Among the industrial by-products, a source of caffeine is represented by spent coffee grounds (from coffee bars), which still have an amount of caffeine in the range of 5.99–11.50 mg/g of dry matter [189].

Another source of methyl-xanthines (including caffeine and theobromine) are cocoa shells [96,190]. This is of particular interest, considering that the high amount of cocoa bean shell produced per year is generally disposed as waste and underutilized as fuel for boilers, animal feed, or fertilizer [191].

Another class of alkaloids, extremely relevant in view of waste valorization, is represented by glycoalkaloids, mainly produced by plants of the Solanaceae family. The major components of the glycoalkaloid family are α-solanine and α-chaconine found in potato plants (*Solanum tuberosum* L.), and solasonine and solamargine found in eggplants (*Solanum melongena* L.), whereas α-tomatine and dehydrotomatine are spirosolane-type glycoalkaloids found in tomato plants (*Lycopersicon esculentum* Mill.) [185].

These compounds are agrochemically important, in fact, their defensive role in plants makes many of them (i.e., α-tomatine, α-chaconine, α-solanine, and various *Solanum* spp. extracts) endowed with insecticidal activity against various insect species. In particular, both α-chaconine and α-solanine decrease insect feeding, delay their development, affect reproduction, and alter insect enzyme activity [185].

Although the majority of the toxicity studies have been focused on tomatoes and potatoes due to the economic importance and availability of these species, acute toxicity to insects has also been reported in plant extracts belonging to other genera, such as *Piper*, *Datura*, and *Withania* [185]. Notably, from *Datura stramonium* L. it is possible to extract a great variety of bioactive alkaloids, saponins, sterols, and polyphenols. This plant, as well as several other plants containing alkaloids, is often considered an agricultural waste [155], since it is invasive and its presence in cultivated fields is undesired.

Potato peels, a by-product of the industrial production of potato fries, chips, and flour, are a significant part of the annual worldwide production of about 1.3 billion tons of food waste [192]. In this context, alkaloids extracted from potato peels proved to be antioxidant [193] and antiprotozoal against pathogenic *Trichomonad* strains that infect humans, farm animals, and felines [192].

Alkaloids from plant wastes have been also tested for biological activities eventually useful for human healthcare, for instance, tomato (*L. esculentum*, Solanaceae) leaves alkaloids proved promising for Alzheimer disease treatment [194].

## 5. Extraction Techniques

### 5.1. Conventional Extraction Procedures and New Prospective for Solvents

Natural products are characterized by great diversity, which implies the necessity to develop specific extraction methods according to the starting raw material and selected metabolite(s). Generally, the raw matrix is subjected to an air drying process, dried in an oven or freeze-dried, and subsequently ground to create a homogeneous sample before the extraction [195,196,197]. Sometimes, before polyphenol extraction, the matrix is defatted with a non-polar solvent like n-hexane [70].

One of the most common extraction methods is solid–liquid extraction using water or organic solvents [65,84]. In some cases the extraction is performed through a Soxhlet extractor, which allows an efficient recycle of the solvent, which can be used in small amounts to extract a significant quantity of plant material [195,198].

If the starting material is a liquid, as for wastewaters, a liquid–liquid extraction, for instance, using ethyl acetate, is preferable [70,71]. Liquid/liquid partition has often been employed also to extract alkaloids and terpenes [199].

Sometimes the extraction can be facilitated by acidic or basic conditions under heating, as in the case of tannins, which can be extracted by maceration under reflux, using aqueous solvents, slightly alkaline at 85 °C. In particular, hydrolysable tannins are well extracted using a blend of 1% NaOH for 240 min, while condensed tannins are extracted with a blend of 1% Na_2_SO_3_ for 960 min [92].

Essential oils are obtained by stem distillation, which extracts preferentially volatile monoterpenes, while resins, which are generally more enriched in diterpenes, are extracted using organic solvents.

However, toxic solvents, like n-hexane or methanol, are unsuitable to extract products conceived for food or health uses. An option is to replace methanol with ethanol, which makes safer and “greener” the process. For example, an excellent polyphenol yield is obtained thought maceration in an hydro-alcoholic blend of 50 or 60% ethanol for 30 min at a temperature between 60 °C and 80 °C, under reflux or simply under continuous stirring at room temperature [61,189].

However, in search of more efficient and environmental friendly alternatives to extract high-value molecules, ionic liquids (ILs) and natural deep eutectic solvents (NADES) have been proposed [200].

ILs are ionic species (organic salts), fluids or solids at room temperature, consisting of an organic cation (i.e., ammonium, imidazolium, pyridinium, phosphonium) and an anion (i.e., bromide, chloride, tetrafluoroborate, hexafluorophosphate). Due to their ionic nature, ILs possess negligible vapor pressure and high solvation ability, and they offer a wide spectrum of extraction abilities and selectivity [201]. Compounds such as flavonoids, alkaloids, phenolics, terpenoids, phenylpropanoids, and polysaccharides have been successfully extracted by ILs [202]. Moreover, recently, the possibility of using aqueous solutions of ILs instead of their pure forms led to a substantial improvement in their extraction efficiency and cost reduction. These solvents have already been used for the extraction of waste from natural products. For instance, solutions of surface-active ILs in water were used to efficiently extract triterpenic acids from apple peels [203], oleanolic acid from *O. europaea* [138], anthocyanins from grape pomace and peel of eggplant [204,205].

Natural deep eutectic solvents (NADES) are considered as a specific class of liquids present in living cells, where they play an important role in biosynthesis, transport, and storage of compounds with intermediate polarity [206]. NADES are composed of hydrogen bond donors (HBDs) and hydrogen bond acceptors (HBAs) mixed together. The usual HBAs are nontoxic quaternary ammonium salts or amino acids, while HBDs are organic acids or carbohydrates. Alcohol, amine, aldehyde, ketone, and carboxylic groups can be used as both HBAs and HBDs. There are a huge number of natural metabolites, which can be combined to prepare NADES, making the latter a high versatility tailor-made class of solvents. NADES have numerous favorable properties, they are liquid state within a wide temperature range, manifest chemical and thermal stability, are non-flammable and non-volatile, nontoxic as well as having sustainable “green” properties. On this basis, NADES have been used to extract a wide range of natural compounds, including phenolics, alkaloids, saponins, anthraquinones, terpenoids, polyunsaturated fatty acids, and photosynthetic pigments [207,208]. NADES and their perspectives in the agri-food sector were extensively reviewed by Mišan et al. [206]. Regarding the employment of NADES in the extraction of plant-driven industrial wastes, they have been used to efficiently obtain phenols from by-products of the olive oil industry, and the onion, tomato, potato, orange, and pear canning industries [209,210].

### 5.2. Non-Conventional Extraction Methods

Different extraction methods have been proposed in order to shorten, improve, and obtain greener procedures for natural metabolite extraction. Several works have been dedicated to summarize these techniques and their use to extract bioactive metabolites from natural wastes [211,212]. Ultrasonic-assisted extraction (UAE) is an effective extraction technique for a wide range of compounds from different types of matrices. Due to the cavitational effects leading to cell wall disruption the release of the target compound(s) from the biomass is favored [213]. In addition, ultrasounds also use the oxidative energy of radicals created during sonolysis to make more efficient the extraction process [214].

This results in a shortening of the extraction process with low consumption of solvents and high product yields. This method has been used to efficiently extract different classes of metabolites from several plants [213].

Microwave-assisted extraction (MAE) reduces extraction time with a minor consumption of solvents and minimum degradation of target compounds. It is based on microwave energy, which heats the solvents and increases the internal pressure inside the cell, helping the disruption of the cellular wall and the release of active compounds to the solvent [215]. Combined with inert atmosphere to avoid polyphenol degradation, this is a sustainable technique [216] that allows to work at high temperature (150 °C), obtaining very good yields of extracts endowed with a high antioxidant power [217]. This method can be optimized using a solution at pH 12, which facilitates the extraction of polyphenols [195].

Supercritical fluid technology (SFT) is based on supercritical fluids, among them the most used is supercritical carbon dioxide (CO_2_), generated by increasing the pressure and the temperature of the liquid/gas above the critical point. These fluids have liquid-like solvent power and gas-like diffusivity, resulting optimal to extract compounds from plant matrices, and yield solvent-free extracts by the reduction of CO_2_ pressure, which allows to easily remove it. This technique has been extensively applied to extract plant metabolites from waste. For instance, STF was used to efficiently extract tocopherols and carotenoids rich oil guava seeds, which are endowed with good antioxidant activity [218].

Supercritical fluid extraction (SFE) was proven not suitable for polyphenol extraction [198], however it could be useful in a first phase where fatty acids are removed from the matrix, and polyphenols might be extracted subsequently with the help of a co-solvent [197].

Among the green technologies, pressurized liquid extraction (PLE), also called accelerated solvent extraction (ASE), is a fully automated technique that combines high temperature and pressure with the use of liquid solvents. This technique proved efficient to extract different matrices such as food [219] medicinal plants [220], and environmental samples [221]. PLE improves extraction yield, significantly reducing time and solvent consumption, [222] and it can be used to extract molecules of different polarity, such as phenolic compounds, carotenoids, and essential oils [222]. An optimized PLE protocol for the extraction of water-soluble molecules is represented by hydrothermal extraction, which uses water at high temperature and high pressure and has been employed to extract spent coffee grounds [223].

## 6. Trends in Publications Focused on Plant Wastes Valorization

**Figure 5:**
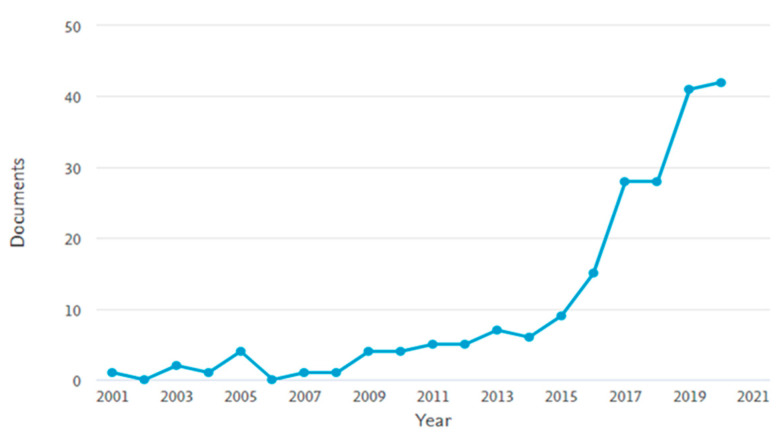
Trend of scientific publications from 2001 to 2020 reported by Scopus using as a query string: TITLE-ABS-KEY (plant AND by-products AND waste AND valorization).

The number of scientific publications dealing with the valorization of waste derived from plants have notably increased in the last three years (Figure 5) [224]. This trend reflects the rising global interest in circular economy and requalification of neglected plant matrices.

This review carried out a bibliographic survey, taking into account 99 scientific articles on this topic published from 2006 to 2020. Table 1 summarizes the information about waste type, its plant source, industrial sector of provenience, contained secondary metabolites, reported bioactivity, and potential use for its valorization. The survey included 64 plants generating wastes, mainly derived from the food and beverage industries, followed by the herbal, agriculture, and forestry industries, and only one report was dedicated to a plant waste from the perfume industry. Moreover, according to this survey, wastes from 16 plant species originated from more than one industrial sector. This is easily understandable taking into account that the majority of the industrial products undergo different steps in the production chain. For example, different kinds of wastes are generated from *Castanea sativa* cultivated for nuts production, and these wastes originate at different level of the production chain. In particular, spiny burs are the main agricultural residue from tree cultivation, while husks are discarded by the food industry when producing chestnut flour. The beverage industry generates also a considerable amount of wastes/by-products from plant origin, most prominent among them fruit pomace and or/peel. Nevertheless, a conspicuous number of wastes are also produced during the previous steps of the supply chain, namely, the fruit production itself leads to several agricultural residues. An example is provided by the wine production chain, where grape pomace is the main final waste, even though the cultivation of *Vitis vinifera* L. itself generates residuals like green prunings. In this case, as highlighted also by our survey, due to their content in polyphenols, flavonoids, and tannins, grape pomace and skin are strongly valorized by-products (12 publications were focused on the requalification of these wastes). However, as pointed out by Acquadro et al. [225], green pruning might be exploited for its phenolic content.

Focusing on the specific classes of SMs considered in the examined papers, polyphenols emerged as the most investigated one. Out of 64 plants mentioned in the reviewed articles, the generic presence of polyphenols was reported in 31 of them. Moreover, several investigations were focused also on specific polyphenol sub-classes, such as flavonoids (found in 21 plants) and phenolic acids (found in 18 plants).

Conversely, terpenes and alkaloids resulted as less investigated; in fact, they were mentioned as important compounds in only 12 and 7 plant species, respectively. In the context of waste valorization, the main bioactivity related to polyphenols was the in vitro antioxidant activity, which is common also to terpenes [29] and alkaloids [189]; this encourages further works dedicated to these latter classes of plant metabolites, often contained in wastes/by-products.

**Figure 6:**
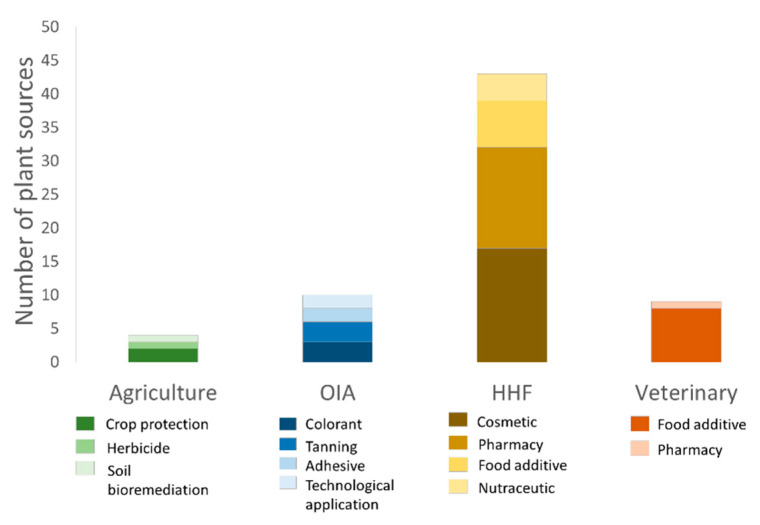
Number of plant matrices investigated for potential application in four different areas: agriculture, OIA (Other Industrial Applications), HHF (Human Healthcare and Food), and veterinary science. The graphic summarizes the results of our bibliographic survey (from 2006 to 2020).

According to this bibliographic survey, terpene saponins resulted as the less explored metabolite from plant waste matrices. Their presence was, in fact, reported only in waste constituted by onion skin and *A. sisalana* leaves. As mentioned above, saponins have numerous applications and bioactivities, among them their possible use in soil bioremediation. Moreover, this survey pointed out that only few waste matrices were studied for their potential use in the agriculture sector (Figure 6).

In particular, *Anacardium occidentale* L. nut shells and *Castanea sativa* burs proved good sources of molecules endowed with pesticide and anti-fungal properties, respectively. On this basis they could find application as crop protection agents. Spent coffee ground showed allelopathic activity, which is useful for herbicide products.

As it is evident in Figure 6, the majority of plant wastes were valorized proposing potential exploitation in the area here called “human healthcare and food” (HHF) (including cosmetic, pharmaceutical, nutraceutical, and food additive use). However, since plant SMs are endowed with a wide range of chemical and biological properties (as summarized in this review), it is expected that, following the actual trend, wastes and by-products of plant origin will be increasingly investigated also for their potential use in other areas of application, especially in agriculture, considering the need to implement “greener” practices in this sector.

## Figures and Tables

**Table 1:** Summary of the results emerging from the survey. The table reports: plant species from which the waste is generated, the industrial sector manufacturing the plant material, the nature of the produced waste, the class of secondary metabolites found in the waste material, the bioactivity possessed by waste extract(s), the potential use for waste valorization, and the corresponding reference source of all the information reported in a line. If no biological activity was investigated, or no potential valorization in a specific field was proposed, the wording “not specified” (n. s.) was inserted in the corresponding cell.

Plant Species ^1^	Waste Producer Industry	Waste Type	Contained Secondary Metabolites	Reported Bioactivity	Potential Use for Waste Valorization	References
*Acacia mangium* Willd.	Forestry	bark	tannins	n. s.	leather tanning	[56]
*Aesculus hippocastanum* L.	Herbal industry	wastewaters	flavonoids	n. s.	n. s.	[79]
*Agave sisalana* Perrine	Fiber Industry	leaves	saponins	n. s.	cosmetic, pharmacy	[151]
					soil bioremediation	
*Allium cepa* L.	Food	peel	flavonoids, saponins	anti-obesity	pharmacy	[44]
				surface activity	food additive	[152]
		pomace		photoprotection	cosmetic	[226]
*Allium sativum* L.	Food	n. s.	flavonoids	photoprotection	cosmetic	[226]
*Aloe barbadensis* Mill.	Agriculture	roots	anthraquinones	antiviral	pharmacy	[227,228]
*Anacardium occidentale* L.	Food	nut shell liquid (CNSL), testa, cashew apple, and cashew apple bagasse	phenolic acids, alkaloids, tannins	pesticide, larvicide, anti-termite, dyes, anti-cancer, anti-bacterial, antioxidant, neurotransmitter, etc.	colorant	[229]
					crop protection	
					pharmacy	
*Ananas comosus* (L.) Merr.	Food	core and skin	polyphenols	antioxidant in vitro	food additive	[230]
*Arachis hypogaea* L.	Food	skin and husks	phenolic acids, flavonoids polyphenols	antioxidant in vitro	feed additive	[231]
				antibacterial	pharmacy	
*Aronia melanocarpa* (Michx.) Elliot	Beverage	pomace	flavonoids	antioxidant in vitro	feed additive	[74]
			phenolic acids			
*Camellia sinensis* (L.) Kuntze	Food	black tea waste	polyphenols	antioxidant in vitro	cosmetic, food additive, pharmacy	[97]
				antibacterial		
*Castanea sativa* Mill.	Agriculture and Food	burs	polyphenols	fungicide, enzyme inhibitor and antioxidant in vitro	cosmetic, crop protection, food additive, pharmacy	[86,87,88,89,90]
		leaves		enzyme inhibitor, antitumoral and antioxidant in vitro		[86,87,88]
		bud		n. s.		[93]
		husks		enzyme inhibitor		[86,88]
				antioxidant in vitro		
		husks and bark	tannins	n. s.	cosmetic, pharmacy, industrial application	[92,94]
*Citrus* spp.	Food and Beverage	peel and pulp	flavonoids, essential oil, phenolic acid	antioxidant in vitro, anti-inflammatory in vitro, antitumoral	cosmetic, food additive, pharmacy	[27,226,228]
*Citrus* x *bergamia* (Risso) Risso & Poit.	Herbal Industry	peel	flavonoids	antibacterial	pharmacy	[228,232]
*Citrus* x *sinensis* (L.) Osbeck	Beverage	peel	essential oil	n. s.	n. s.	[233]
*Coffea* spp.	Beverage and Food	spent coffee grounds and silverskin	polyphenols and alkaloids	antioxidant in vitro	cosmetic, food additive, nutraceutical, pharmacy	[80,81,84,189]
		spent coffee grounds	n. s.	anti-pollutants		[234]
		spent coffee grounds	polyphenols	allelopathic activity	herbicide	[82]
*Coffea arabica* L.	Food	silverskin	polyphenols	antioxidant in vitro	n. s.	[85]
*Cucumis melo* L.	Food and Beverage	peel	polyphenols	antioxidant in vitro	cosmetic, nutraceutical, food additive, pharmacy	[98]
*Cucurbita* spp.	Food	peel, seeds, and fruits not suitable for human consumption	terpenes and phenolic acids	improvement of meat, milk, or eggs	feed additive	[235]
*Curcuma longa* L.	Agriculture	leaves	terpenes	anti-inflammatory in vivo and in vitro	pharmacy	[236]
*Cynara scolymus* L.	Food	floral stems	polyphenols, flavonoids, tannins	antioxidant in vitro, antibacterial, anti-denaturating protein, antidiabetic in vivo and anti-hyperlipidemic in vivo	nutraceutical	[237]
	Agriculture	bracts of the heads			cosmetic	[75]
*Datura stramonium* L.	Agriculture	leaves and flowers	terpenes	antibacterial	pharmacy	[155]
*Daucus carota* L.	Beverage	pomace	terpenes	antioxidant in vitro	feed additive	[74]
*Foeniculum vulgare* Mill.	Herbal Industry	seeds solid residue from distillation	flavonoids and polyphenols	antibacterial and antioxidant in vitro	food additive	[228,238]
	Agriculture	leaves, inflorescence, and pseudo stems	terpenes	antioxidant in vitro	n. s.	[137]
*Fragaria* spp.	Beverage	pomace	flavonoids	antioxidant in vitro	feed additive	[74]
*Humulus lupulus* L.	Beverage	leaves and flowers	terpenes	antibacterial	pharmacy	[155]
*Hyssopus officinalis* L.	Herbal Industry	solid distillation residue	phenolic acids	antioxidant in vitro	food additive	[228,239]
*Ilex paraguariensis* A. St.-Hil.	Beverage	exhausted leaves	terpenes, flavonoids, polyphenols	antioxidant in vitro	n. s.	[240]
*Juglans* spp.	Agriculture and Food	husks	tannins, flavonoids, terpenes, phenolic acids, anthraquinones, naphthoquinones	removal of hazardous materials, antioxidant in vitro, antibacterial, anti-platelet, cytotoxic	cosmetic, pharmacy	[241]
					colorant	
*Larix kaempferi* (Lamb.) Carrière	Forestry	bark	flavonoids	enzyme inhibitor	cosmetic	[99]
*Lavandula* x *intermedia* Emeric ex Loisel.	Perfume Industry	solid distillation residue	flavonoids, polyphenols, phenolic acids	antioxidant in vitro	n. s.	[228,242]
*Lycopersicon esculentum* Mill.	Agriculture	leaves	polyphenols and alkaloids	enzyme inhibitor	pharmacy	[194]
*Lycopersicon lycopersicum* (L.) H. Karst.	Food	peel	polyphenols	antioxidant in vitro	cosmetic, food additive, pharmacy	[75]
*Malus* spp.	Beverage	pomace	flavonoids and polyphenols	antioxidant in vitro, additive	cosmetic, food additive, pharmacy	[74,75,217]
*Malus domestica* (Suckow) Borkh.	Food and Beverage	pomace	flavonoids and phenolic acids	antioxidant in vitro, photoprotection	cosmetic, feed additive	[74,226]
*Mangifera indica* L.	Food	pomace	polyphenols	photoprotection	cosmetic	[226]
*Ocimum basilicum* L.	Food and Herbal Industry	oil distillation wastewaters	polyphenols	antioxidant in vitro	cosmetic, food additive, nutraceutical, pharmacy	[78,228,243]
*Olea europaea* L.	Food	distillation wastewater, pomace, leaves	polyphenols	antioxidant in vitro, antibacterial, decrease lipolysis	cosmetic, food additive, nutraceutical, pharmacy	[69,70,71,153,226,244]
		leaves, fruit milled waste	terpenes	antiallergic	nutraceutical, pharmacy	[153,154]
		pomace	polyphenols	antioxidant in vitro, improvement of gut microbiota	feed additive	[68]
*Pinus* spp.	Forestry	resin	terpenes	improve thermal stability of bio-based materials	technological application	[149]
				organocatalyst		[150]
*Pinus pinaster* Aiton	Agriculture	bark	tannins	tanning	leather tanning	[95]
*Poliomintha longiflora* A. Gray	Herbal Industry	solid distillation residue	polyphenols, phenolic acids, acids, terpenes	antibacterial and antioxidant in vitro	n. s.	[136,228]
*Prunus avium* (L.) L.	Food	peel and stems	polyphenols	antioxidant in vitro, enzyme inhibitor and photoprotection	cosmetic	[245]
*Prunus cerasus* L.	Food and Beverage	cherry liquor pomace, pomace, and seeds	polyphenols	antioxidant in vitro	cosmetic, nutraceutical	[77,246,247]
		stems, leaves, pomace, and seeds	terpenes	antibacterial	nutraceutical	
*Prunus dulcis* (Mill.) D. A. Webb	Agriculture and Food	husks, skins, and blanching water	polyphenols and flavonoids	antibacterial, antioxidant in vitro	pharmacy, technological application	[248]
*Punica granatum* L.	Food and Beverage	peel, arils, mesocarp, and pulp	polyphenols	antioxidant in vitro, antibacterial, photoprotection	cosmetic	[226,249,250,251,252,253]
*Pyrus* spp.	Beverage	pomace	polyphenols	antioxidant in vitro	cosmetic	[75]
*Quercus suber* L.	Forestry	by-product of bark processing	phenolic acids and tannins	antioxidant in vitro	cosmetic	[254]
*Ribes nigrum* L.	Beverage	pomace	tannins and flavonoids	dyes, antioxidant in vitro	feed additive	[74]
			phenolic acids and flavonoids	enzyme inhibitor, antioxidant in vitro	cosmetic	[48,255]
*Salvia officinalis* L.	Herbal industry and Food	oil distillation wastewaters	polyphenols	antioxidant in vitro	n. s.	[78]
*Salvia officinalis* subsp. *Lavandulifolia* (Vahl) Gams	Herbal Industry	solid distillation residue	phenolic acids	antioxidant in vitro	cosmetic, food additive	[228,256]
*Salvia rosmarinus* Spenn.	Herbal Industry	solid distillation residue	polyphenols and phenolic acids	antioxidant in vitro	food additive	[228,257]
		oil distillation wastewaters	terpenes and polyphenols	antioxidant in vitro	n. s.	[78]
*Santolina chamaecyparissus* L.	Herbal Industry	solid distillation residue	phenolic acids	antioxidant in vitro	pharmacy	[228,239]
*Satureja montana* L.	Herbal Industry	solid distillation residue	phenolic acids	antioxidant in vitro	pharmacy	[228,239]
*Solanum lycopersicum* L.	Food	pomace	terpenes	antioxidant in vitro, photoprotection, chemoprevention	cosmetic, nutraceutical	[29,226]
*Solanum tuberosum* L.	Food	peel	polyphenols and alkaloids	antioxidant in vitro	n. s.	[193]
		peel		anti-trichomonads	pharmacy	[192]
*Solidago virgaurea* L.	Herbal industry	dried herb	polyphenols	antioxidant in vitro	cosmetic, food additive	[75]
*Sophora flavescens* Aiton	Herbal Industry	all plants	flavonoids and alkaloids	anti-inflammatory in vitro	pharmacy	[258]
	Agriculture	seeds	alkaloids	antioxidant in vitro and antibacterial	n. s.	[259]
*Theobroma cacao* L.	Food	bean shell	phenolic acids	antioxidant in vitro	food additive	[195]
			polyphenols and alkaloids		n. s.	[190]
			flavonoids and alkaloids		n. s.	[96]
*Thymus mastichina* (L.) L.	Herbal Industry	solid distillation residue	phenolic acids	antioxidant in vitro	n. s.	[228,256]
*Ugni molinae* Turcz	Food and Herbal Industry	seeds	polyphenols	antibacterial	n. s.	[260]
*Vaccinium myrtillus* L.	Beverage	pomace	flavonoids	dyes	colorant	[76]
*Vitis labrusca* L.	Beverage	pomace	polyphenols	n. s.	nutraceutical	[250,261]
*Vitis vinifera* L.	Agriculture	green pruning	polyphenols	antioxidant in vitro	cosmetic, nutraceutical, pharmacy	[225]
	Beverage	seeds, pomace, and stems	flavonoids, polyphenols	antioxidant in vitro, antibacterial		[225,250,262,263,264,265]
		pomace	flavonoids, polyphenols	antioxidant in vitro, photoprotection, cholesterol-lowering activities		[61,63,226,250,251,261,264,266]
		pomace	polyphenols, tannins, flavonoids, saponins	antioxidant in vivo, anthelmintic	feed additive, pharmacy	[62,267]
		pomace	tannins	adhesive properties	adhesive	[64]
*Zea mays* L.	Agriculture	maize bran	phenolic acids	n. s.	n. s.	[100]

^1^ Plant scientific names have been updated following the World Checklist of Vascular Plants (WCVP 2020) [268].

## Data Availability

The data presented in this study are openly available in this article.
